# HBXIP overexpression is correlated with the clinical features and survival outcome of ovarian cancer

**DOI:** 10.1186/s13048-017-0322-7

**Published:** 2017-04-07

**Authors:** Yixuan Wang, Jie Sun, Nan Li, Shuanlong Che, Tiefeng Jin, Shuangping Liu, Zhenhua Lin

**Affiliations:** 1 Key Laboratory of Natural Resources of Changbai Mountain & Functional Molecules, Yanbian University, Yanji, 133002 Jilin China; 2 Department of Pathology & Cancer Research Center, Yanbian University Medical College, Yanji, 133002 Jilin China

**Keywords:** Ovarian cancer, Hepatitis B virus X-interacting protein, Immunohistochemistry, Survival analysis

## Abstract

**Background:**

Accumulated evidence has demonstrated that Mammalian hepatitis B X-interacting protein (HBXIP) has broad roles in cancer. Although HBXIP is associated with a variety of cancers, the HBXIP protein expression level and its clinical significance in ovarian cancer have not yet been determined. The aim of this study is to investigate the association between HBXIP expression and the clinicopathological features of ovarian cancer patients to determine whether HBXIP may be correlated with a poor prognosis in ovarian cancer patients.

**Methods:**

HBXIP protein expression was assessed in a well-characterized series of ovarian cancer tissue samples (*n* = 120) with long-term follow-up, using immunohistochemistry to determine the location pattern and expression of HBXIP in ovarian cancer. The localization of HBXIP was detected in SKOV-3 ovarian cancer cells using immunofluorescence (IF) staining. The relationship between high HBXIP expression and the clinicopathological features of ovarian cancer patients was analyzed by Chi-square and Fisher’s exact test. Overall survival (OS) rates of all the ovarian cancer patients were calculated using the Kaplan-Meier method, and univariate and multivariate analyses were performed using the Cox proportional hazards regression model.

**Results:**

IF staining revealed strongly positive signals for HBXIP in both cytoplasm and nucleus, but mainly in the cytoplasm of SKOV-3 ovarian cancer cells. High HBXIP expression was predominantly observed in ovarian cancer tissues but not the adjacent non-tumor ovarian tissues. The strongly positive rate of HBXIP expression was 60.0% (72/120) in ovarian cancer and was significantly higher than in adjacent non-tumor tissues (17.4%, 4/23) (*P* = 0.000). High HBXIP expression was positively correlated with the occurrence of lymph node metastases (*P* = 0.025), histological grade (*P* = 0.036) and clinical stage (*P* = 0.003). The patients with high HBXIP expression had lower overall survival (OS) rates. Moreover, multivariate analysis indicated that HBXIP, in addition to the clinical stage, was a significant independent prognostic factor in patients with ovarian cancer.

**Conclusions:**

High-level expression of HBXIP is associated with the progression of ovarian cancer and may be an effective biomarker for poor prognostic evaluation as well as a potential molecular therapy target for ovarian cancer patients.

**Electronic supplementary material:**

The online version of this article (doi:10.1186/s13048-017-0322-7) contains supplementary material, which is available to authorized users.

## Background

Ovarian cancer is one of the common lethal malignancies in women and the most common cause of gynecologic cancer deaths [1]. Most patients are diagnosed in advanced stages, so clinical treatment and prognosis are not satisfactory. Currently, clinical characteristics in ovarian cancer, such as the FIGO stage, tumor type, the presence of peritoneal metastases, lymph node status, and morphological characteristics, are the most important prognostic factors [2]. Therefore, early diagnosis and early treatment are the most effective methods of prevention and treatment of ovarian cancer, and efficient screening methods can significantly reduce the mortality rate of patients and improve the prognosis.

Mammalian hepatitis B X-interacting protein (HBXIP) is a conserved 18 kDa protein, which was originally identified due to its interaction with the C terminus of the hepatitis B virus X protein [3]. HBXIP was shown to interact with the hSuv3 protein, which encodes an NTP-dependent DNA/RNA DE*X*H box helicase predominantly localized in mitochondria [4]. Furthermore, HBXIP also functions as a regulatory component in amino acid-induced activation of mTORC1 in cell growth [5]. Much evidence has demonstrated that hepatitis B virus X-interacting protein plays broad roles in several types of cancers, including breast cancer, ovarian cancer and hepatocellular carcinoma. Previous studies reported that HBXIP was able to promote breast cancer cell proliferation and migration via activation of transcription factors [6–11]. It also promotes the migration of breast cancer cells through modulating microtubule acetylation mediated by GCN5 [12]. Similarly, the oncoprotein HBXIP promotes the migration of ovarian cancer cells through the upregulation of S-phase kinase-associated protein 2 by Sp1 [13]. In addition, HBXIP functions as a cofactor for survivin and serves as a link between the cellular apoptosis machinery and a viral pathogen involved in hepatocellular carcinogenesis [14]. HBXIP increases hepatoma HepG2 cell-induced endothelial cell migration, proliferation, and angiogenesis, and plays an important role in tumorigenesis by enhancing angiogenesis in HCC [15]. Similarly, HBXIP can markedly enhance angiogenesis and growth of breast cancer through upregulating the expression and secretion of FGF8 and VEGF [16]. Therefore, the abnormal expression of HBXIP may facilitate carcinogenesis and tumor progression.

Although HBXIP is associated with a variety of cancers, the HBXIP protein expression level and its clinical significance in ovarian cancer have not yet been determined. The aim of this study is to investigate the association between HBXIP expression and the clinicopathological features of ovarian cancer patients to determine whether HBXIP may be correlated with poor prognosis in ovarian cancer patients. We used immunohistochemistry (IHC) to identify HBXIP in ovarian cancer tissues and examined the association of HBXIP protein expression with the clinicopathological features of ovarian cancer. We also assessed the prognostic value of high HBXIP expression in patients with ovarian cancer.

## Methods

### Ethic statement

This research complied with the Helsinki Declaration and was approved by the Human Ethics Committee and the Research Ethics Committee of Yanbian University Medical College. Patients were informed that the resected specimens were stored by the hospital and potentially used for scientific research and that their privacy would be maintained. Follow-up survival data were collected retrospectively through medical record analyses.

### Clinical samples

A total of 143 human ovarian tissue samples were used for this study, including 120 ovarian cancer tissues, and 23 adjacent non-tumor tissues. These tumors were selected randomly from patients undergoing surgery between 2004 and 2009 and stored in the Tumor Tissue Bank of Yanbian University Medical College. All tissues were routinely fixed in 10% buffered formalin and embedded in paraffin blocks. The study protocol was approved by the institutional review board of Yanbian University Medical College. The pathological parameters, including age, tumor size, occurrence of distant metastases, clinical stage, differentiation, occurrence of nodal metastases and survival data, were carefully reviewed in all 120 ovarian cancer cases.

All patients were female between the ages of 42 to 78 years. A total of 120 patients, 65 cases, were 55 years old or over, and 55 cases were patients below 55 years of age. All cases were confirmed by pathological examination. For the tumor sizes, 59 cases were less than or equal to 5 cm, and 61 cases were greater than 5 cm. TNM staging was assessed according to the staging system established by the American Joint Committee on Cancer (AJCC). Of the 120 ovarian cancer patients, 39 cases were stages I-II, while 81 cases were stages III-IV. For the histological grade, 41 cases were defined as grade-1, 28 cases were grade-2, and 51 cases were grade-3. In addition, 43 cases had distant metastases and 77 cases had no distant metastases. Additionally, 60 cases had lymph node (LN) metastases, and 60 cases had no LN metastases. None of the patients received radio-chemotherapy before surgery. The 120 ovarian cancer patients had been followed for 10 years or until death. The actual survival months of ovarian cancer patients are presented in Additional file 1: Table S. In this study, in 23 cases, adjacent non-tumor lung tissues were collected from the cancer resection margin.

### Immunohistochemical (IHC) staining

HBXIP was purchased from the Dako ProteinTech Group (#14492-1-AP, ProteinTech, USA) of the United States of America. The following steps were performed using the EnVision method. Briefly, 4 μm-thick tissue sections were dewaxed in the standard manner, followed by gradient ethanol hydration, antigen retrieval in hot citrate buffer and washing in PBS buffer. Then, a 1:500 dilution of rabbit HBXIP antibody was added and the sample was incubated overnight at 4 °C. The sample was labeled with horseradish peroxidase anti mouse secondary antibodies at room temperature for 45 min, followed by DAB coloration, counterstaining, and neutral gum sealing. PBS solution was used as the negative control.

### Evaluation of the IHC staining

Two pathologists (Lin Z & Che S) who did not possess knowledge of the clinical data examined and scored all tissue specimens. In case of discrepancies, a final score was established by reassessment by both pathologists on a double-headed microscope. Briefly, the IHC staining for HBXIP was semi-quantitatively scored as ‘-’ (negative) (no or less than 5% positive cells), ‘+’ (5–25% positive cells), ‘++’ (26–50% positive cells) and ‘+++’ (more than 50% positive cells). A cytoplasmic/nuclear expression pattern was considered positive staining. Tissue sections scored as ‘++’ and ‘+++’ were considered strong positives (high-level of expression) for HBXIP. For survival data analysis, ‘++’ or ‘+++’ scored samples were considered as high HBXIP expression and ‘-’or ‘+’ scored samples were considered as low HBXIP expression.

### Immunofluorescence (IF) staining analysis

IF staining was used to detect the sub–cellular localization of HBXIP in SKOV-3 ovarian cancer cells. All steps were performed at RT. SKOV-3 cells were grown on coverslips to 70–80% confluence, then fixed with 4% paraformaldehyde for 10 min. After 24 h, cells were permeabilized with 0.5% TritonX-100 for 10 min. After blocking with 3% bovine albumin fraction V (A8020, Solarbio, Beijing, China) for 1 h, the slides were quickly and gently washed with PBS. The cells were then incubated with the HBXIP antibody (1:1000) at 4 °C overnight, followed by incubation with Alexa Fluor 488 goat anti-rabbit IgG (H + C) (A11008, 1:1000, Invitrogen, USA) for 1 h. After washing with PBS, cells were counterstained with 4’,6-diamidino-2-phenylindole (DAPI) (C1006, Beyotime, Shanghai, China) and the coverslips were mounted with Antifade Mounting Medium (P0126, Beyotime, Shanghai, China). IF signals were visualized and recorded with a BX53 Olympus microscope.

### Statistical methods

All statistical analyses were performed by SPSS17.0 statistical software (SPSS Inc., Chicago, IL, USA). Statistically significant associations between HBXIP expression and clinicopathological parameters were determined by Chi-square (χ^2^) or Fisher’s test. The survival rate was estimated using the Kaplan-Meier method, and differences in survival curves were analyzed using the Log-rank tests. Multivariate analysis was performed using the Cox proportional hazards regression model on all significant characteristics measured for univariate analysis. *P* < 0.05 was considered statistically significant.

## Results

### High expression of HBXIP protein in ovarian cancer

**Fig. 1:**
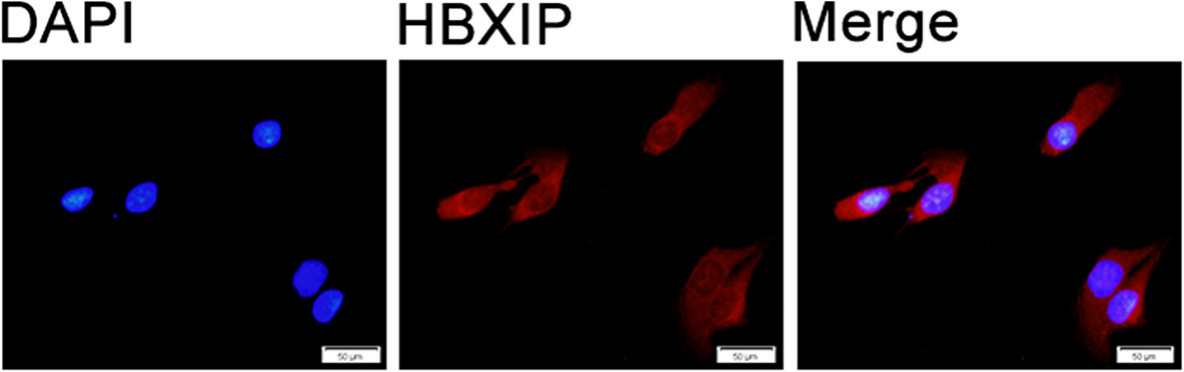
Immunofluorescence staining for HBXIP protein in SKOV-3 ovarian cancer cells. SKOV-3 ovarian cancer cells were immunostained for HBXIP (*red*). Nuclei were visualized by DAPI staining (*blue*). HBXIP protein was localized in both cytoplasm and nucleus, but mainly in the cytoplasm of SKOV-3 ovarian cancer cells

**Fig. 2:**
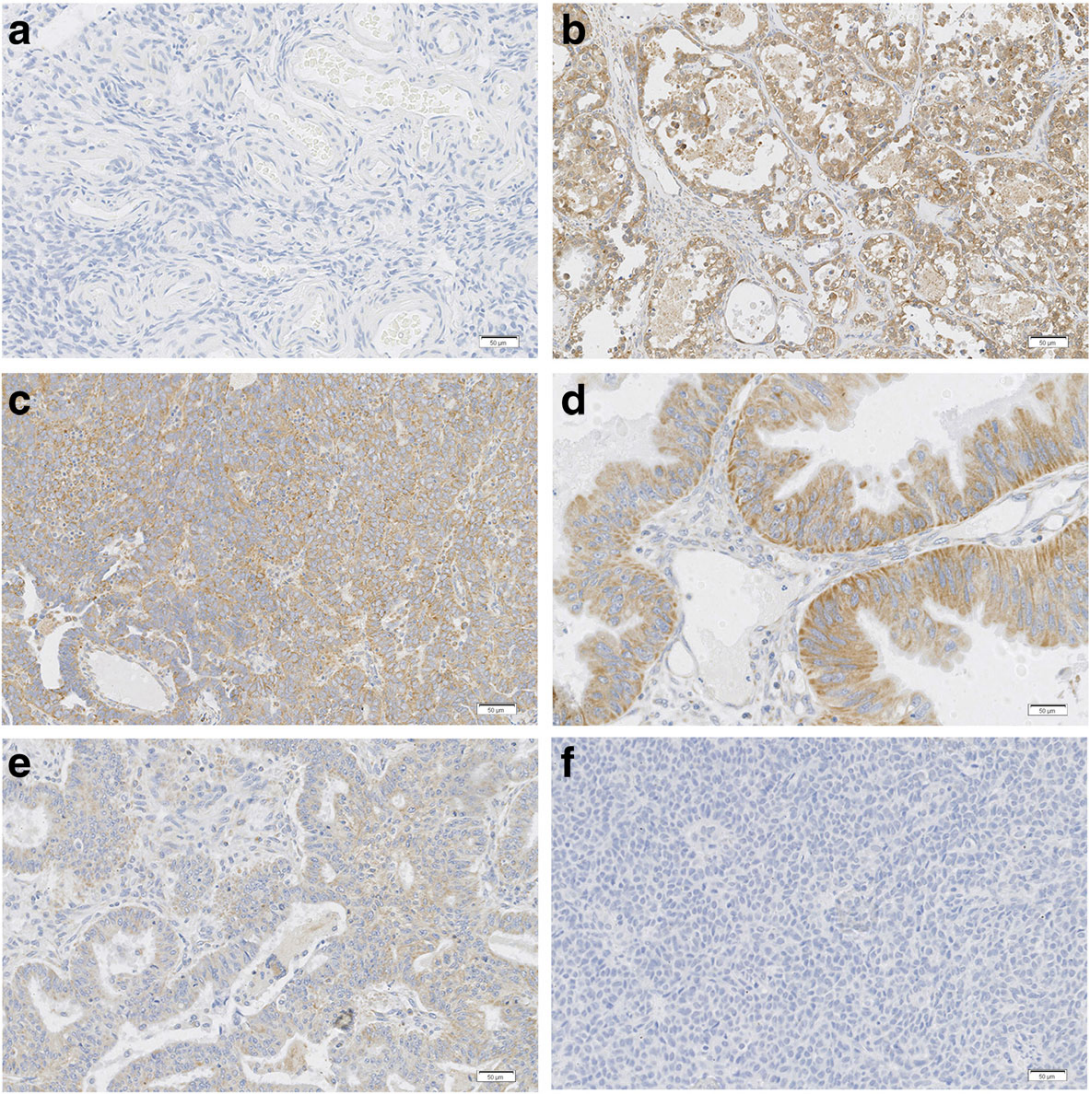
IHC staining of HBXIP protein in ovarian tumor samples. **a** HBXIP protein was negative in adjacent non-tumor ovarian tissues. **b** Diffuse and strong positive HBXIP protein signal in mucinous adenocarcinoma. **c** HBXIP protein was showed diffuse and strong positive staining in serous carcinoma. **d** Diffuse and strong positive HBXIP protein signal in serous carcinoma. **e** Diffuse and strong positive HBXIP protein signal in the endometrioid adenocarcinoma. **f** HBXIP protein staining is negative in granulosa cell tumors. (Original magnification, 200× in **a-f**)

To determine the subcellular localization of HBXIP, IF staining for HBXIP protein was performed in SKOV-3 ovarian cancer cells. IF staining revealed that strongly positive signals for HBXIP were present in both cytoplasm and nucleus, but mainly in the cytoplasm of SKOV-3 ovarian cancer cells (Fig. 1). IHC staining demonstrated that the expression of HBXIP was positive in 101 (84.2%) of the 120 cancer patients and strongly positive in 72 (60.0%) of the 120 patients (Table 1). Further analysis revealed that HBXIP staining was predominantly observed in ovarian cancer tissue but not the adjacent non-tumor ovarian tissues/non-cancerous tissues (*P* < 0.01). As shown in Fig. 2, HBXIP was detected in both cytoplasm and nucleus but primarily in the cytoplasm of cancer cells, with a combined pattern of staining predominating.

**Table 1:** Expression of HBXIP protein in ovarian cancer

Diagnosis	No. of case	HBXIP				Positive case rate (%)	Strong positive case rate (%)
		-	+	++	+++		
Ovarian cancer	120	19	29	37	35	84.2%**	60.0%**
Adjacent non-tumor ovarian	23	15	4	4	0	34.8%	17.4%

Positive rate: percentage of positive cases with +, ++, and +++ staining score

Strongly positive rate (high-level expression): percentage of positive cases with ++ and +++ staining score

**p < 0.01 compared with adjacent normal ovarian

### Correlation between HBXIP expression status and clinicopathological features of ovarian cancer

To evaluate the relationship between HBXIP and ovarian cancer progression, we analyzed the correlation between high HBXIP expression and clinicopathological features of ovarian cancer. As summarized in Table 2, overexpression of HBXIP was significantly correlated with lymph node metastases, histological grade and the clinical stage. However, there were no significant correlations between the expression level of HBXIP and patient age, tumor size or distant metastases in patients with ovarian cancer. For the histological grade, the strongly positive expression of HBXIP was significantly higher in Grade-3 ovarian cancers (72.5%, 37/51) than in Grade-2 (57.1%, 16/28) and Grade-1 (46.3%, 19/41) cancers. Similarly, overexpression of HBXIP showed a correlation with the clinical stage of ovarian cancer. The strongly positive expression rate of HBXIP protein was 69.1% (56/81) in ovarian cancers with an advanced clinical stage (stage III–IV), which was significantly higher than in cases in the early clinical stage (stage I–II) (41.0%, 16/39). Additionally, the strongly positive expression rate of HBXIP was also higher in ovarian cancer with lymph node metastases (70.0%, 42/60) than in cases without metastases (50.0%, 30/60). Taken as a whole, the expression of HBXIP was positively correlated with lymph node metastases, histological grade and clinical stage (Table 2).

**Table 2:** Relationship between HBXIP protein overexpression and the clinicopathological features of ovarian cancer

Variables	No. of case (*n*)	HBXIP strong positive rate (%)	χ^2^	*P* value
Age (years)				
≥ 55	65	36 (55.4%)	1.259	0.262
< 55	55	36 (65.5%)		
Tumor size				
≤ 5 cm	59	37 (62.7%)	0.356	0.551
> 5 cm	61	35 (57.4%)		
M classification				
M0	77	46 (59.7%)	0.006	0.938
M1	43	26 (60.5%)		
Lymph node status				
N0	60	30 (50.0%)	5.000	0.025*
N+	60	42 (70.0%)		
Histological grade				
Grade-1	41	19 (46.3%)	6.629	0.036*
Grade-2	28	16 (57.1%)		
Grade-3	51	37 (72.5%)		
Clinical stage				
I + II	39	16 (41.0%)	8.667	0.003**
III + IV	81	56 (69.1%)		

*p < 0.05 and **p < 0.01

### High HBXIP expression is an independent biomarker of poor prognosis in patients with ovarian cancer

**Fig. 3:**
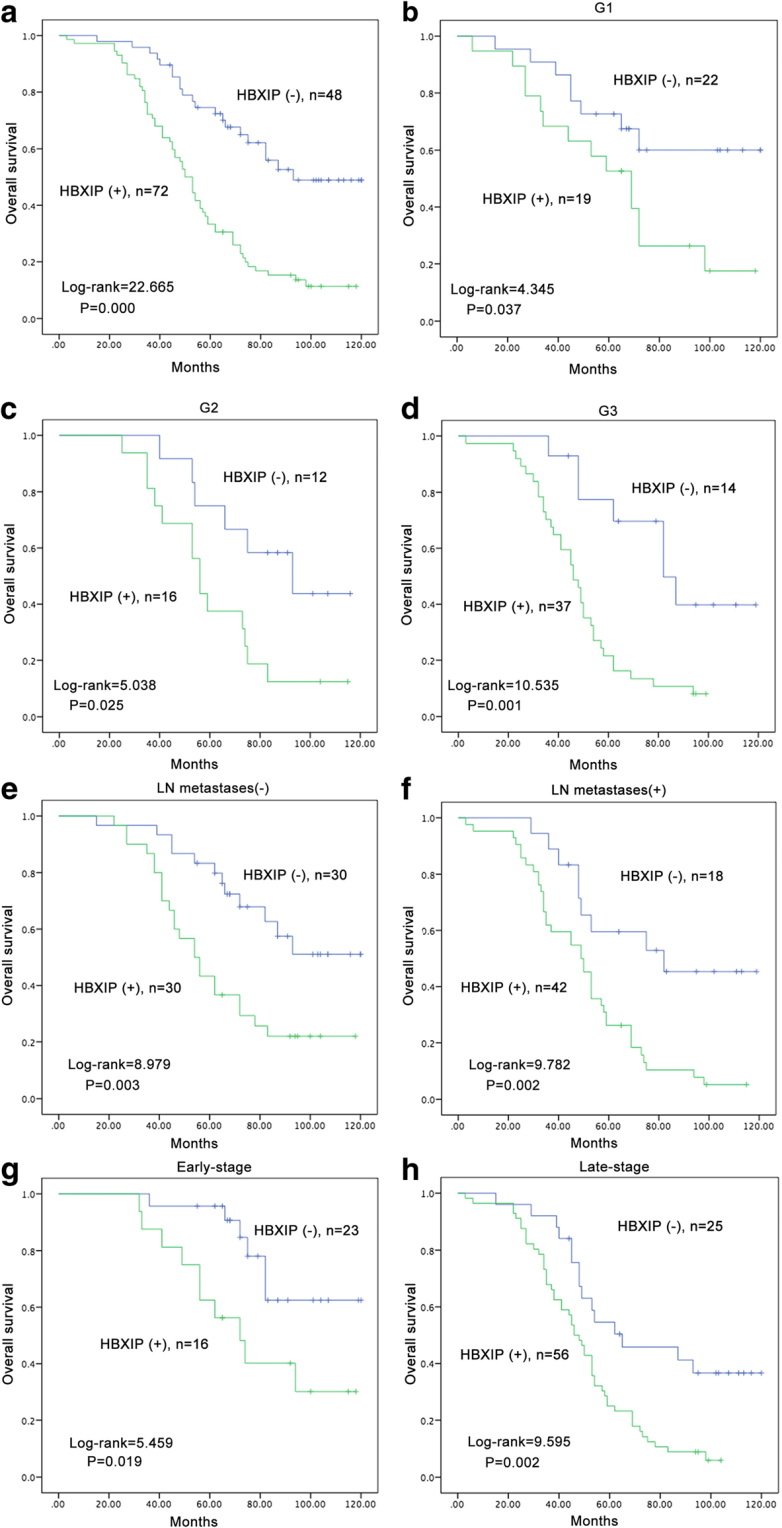
Kaplan-Meier survival curves illustrating the significance of HBXIP expression in ovarian cancer. **a** OS rates of patients with high (*n* = 72) and low (*n* = 48) HBXIP expression. **b**–**d** High HBXIP expression was strongly associated with poor OS in G1 (*n* = 19), G2 (*n* = 16) and G3 (*n* = 37). **e**–**f** High HBXIP expression was strongly associated with poor OS in patients without lymph node metastasis (*n* = 30) and with lymph node metastasis (*n* = 42). **g**–**h** High HBXIP expression was strongly associated with poor OS in early-stage (*n* = 16) and late-stage (*n* = 56). (**a**: Log-rank =22.665, *P* = 0.000; **b**: Log-rank =4.345, *P* = 0.037; **c**: Log-rank = 5.038, *P* = 0.025; **d**: Log-rank = 10.535, *P* = 0.001; **e**: Log-rank =8.979, *P* = 0.003; **f**: Log-rank = 9.782, *P* = 0.002; **g**: Log-rank = 5.459, *P* = 0.019; **h**: Log-rank = 9.595, *P* = 0.002.)

To further substantiate the importance of high HBXIP expression in ovarian cancer progression, we evaluated the prognostic power of HBXIP in determining the OS of 120 ovarian cancer patients using the Kaplan–Meier method. Patients with high HBXIP expression exhibited a lower rate of OS than those with low HBXIP expression (Log-rank = 22.665, *P* = 0.000) (Fig. 3a). Moreover, survival of patients with G1 (Log-rank = 4.345, *P* = 0.037), G2 (Log-rank = 5.038, *P* = 0.025) and G3 (Log-rank = 10.535, *P* = 0.001) ovarian cancer was significantly lower in patients with tumors exhibiting high versus low HBXIP expression (Fig. 3b–d). Similarly, the patients with high HBXIP expression had decreased OS compared to those with low HBXIP expression in cases where LN metastases were not present (−) (Log-rank = 8.979, *P* = 0.003) or in cases when LN metastases were present (+) (Log-rank = 9.782, *P* = 0.002) (Fig. 3e–f). Additionally, ovarian cancer patients with high HBXIP expression had decreased OS compared with those with low HBXIP expression in either early-stage cases (Log-rank = 5.459, *P* = 0.019) or late-stage cases (Log-rank = 9.595, *P* = 0.002) (Fig. 3g–h).

Univariate analysis demonstrated that the occurrence of lymph node metastases (*P* = 0.029), histological grade (*P* = 0.043), clinical stage (*P* = 0.005) and HBXIP expression status (*P* = 0.000) were all significantly associated with OS in patients with ovarian cancer. These data suggest that HBXIP may be a valuable prognostic indicator in ovarian cancer. Multivariate analysis was subsequently performed using the Cox proportional hazards model for all significant variables examined in the univariate analysis. We found that high expression of HBXIP (*P* = 0.002) and clinical stage (*P* = 0.038) were significant independent prognostic factors for survival in ovarian cancer (Table 3).

**Table 3:** Cox regression model analysis of the clinicopathological features in 120 patients with ovarian cancer

Characteristics	B	SE	Wald	HR	95% CI		*P* value
					Lower	Upper	
Univariate							
Age	0.068	0.186	0.133	1.070	0.744	1.539	0.715
Tumor size	0.093	0.185	0.253	1.098	0.764	1.577	0.615
M classification	0.151	0.192	0.620	1.163	0.799	1.693	0.431
LN	0.406	0.185	4.795	1.500	1.043	2.157	0.029*
Grade	0.223	0.110	4.110	1.250	1.007	1.552	0.043*
Clinical stage	0.560	0.197	8.068	1.751	1.190	2.576	0.005**
HBXIP	0.717	0.195	13.575	2.049	1.399	3.000	0.000**
Multivariate							
Grade	0.104	0.116	0.797	1.109	0.883	1.394	0.372
LN	0.221	0.194	1.295	1.247	0.853	1.823	0.255
Clinical stage	0.422	0.204	4.289	1.526	1.023	2.275	0.038*
HBXIP	0.632	0.199	10.058	1.880	1.273	2.778	0.002**

*B* coefficient, *SE* standard error, *Wald* Waldstatistic, *HR* hazard ratio, *CI* confidence interval

*p < 0.05 and **p < 0.01

## Discussion

Mammalian HBXIP is a conserved ~18 kDa protein of unclear function, which was originally identified because of its interaction with HBx, and it is located at human chromosome 1p13.3 [3]. HBXIP sequences are well conserved among mammalian species, with close orthologs found in all vertebrate species where sequence data exists thus far. Overexpression of HBXIP suppresses hepatitis B virus replication in HepG2 cells, in addition to suppressing the transactivation phenotype of HBx [3]. Recent evidence has shown that viral HBx dysregulates the function of cellular HBXIP, causing excessive centrosome production and multipolar mitotic spindles that lead to chromosome segregation defects, thereby creating genetic instability, which is a hallmark of malignancy [17].

Increasing evidence indicates that HBXIP is associated with the regulation of cell proliferation [18]. Moreover, high HBXIP expression has been observed in some cancers. Wang F et al.’s findings indicate that one of the functions of HBXIP involves proliferation regulation in cancer cell lines and in the normal liver cell line, which is related to cell-cycle transition through checkpoint controls at the G0/G1 or G2/M phases and the downregulation of p27 [18]. Li H et al. also found that highly expressed HBXIP accelerates the MDM2-mediated degradation of p53 in breast cancer through modulating the feedback loop of MDM2/p53, resulting in the fast growth of breast cancer cells [19]. Li Y et al. have reported that HBXIP promotes the migration of breast cancer cells through increasing filopodia formation involving MEKK2/ERK1/2/Capn4 signaling [20]. Xu F et al. found that HBXIP promoted the proliferation of breast cancer cells, resulting in the increase of S-phase cells, through upregulating Skp2 via activating transcription factor E2F1 [6]. Importantly, they also reported that HBXIP was able to stimulate the activity of the Skp2 promoter via transcription factor Sp1 thus promoting the migration of ovarian cancer cells [13].

Accumulating evidence demonstrates that glucose metabolism reprogramming and abnormal lipid metabolism are hallmarks of tumorigenesis. In breast cancer, HBXIP can enhance glucose metabolism reprogramming through suppressing SCO2 and PDHA1. It also contributes to abnormal lipid metabolism through LXRs/SREBP-1c/FAS signaling [21, 22]. These findings indicate that HBXIP can be used as a co-activator of transcription factors to upregulate many genes in the development of cancer. However, the molecular mechanism of HBXIP-mediated promotion of tumor proliferation is still unclear; so far, no association of HBXIP expression with the clinicopathological factors in ovarian cancer patients has been reported.

In this study, we incorporated staining intensity and percentage of positive tumor cells for the scoring of HBXIP expression. Our reported results are similar to the trends reported in the literature. Xu’s data showed that the positive expression rate of HBXIP was 75% (60/80) in clinical ovarian cancer tissue samples [13]. Similar to Xu’s studies, our study also indicated that HBXIP staining was predominantly present in ovarian cancer tissue but not the adjacent normal ovarian tissues/non-cancerous tissues [13]. However, they did not analysis the relationship between HBXIP expression and clinicopathological features. In our study, we found that staining of HBXIP was localized in both cytoplasm and nucleus but mainly in the cytoplasm, and these observations were consistent with our IF staining results in SKOV-3 ovarian cancer cells. Moreover, HBXIP expression was found to be related to the histological grade and clinical stage in the 120 ovarian cancer patients. Here, we also found that the patients with high HBXIP expression had lower OS than those with low HBXIP expression, as determined using the Kaplan-Meier method. Moreover, the Cox regression analysis showed that HBXIP was an independent prognostic factor for OS, along with tumor clinical stage.

These statistics indicated that HBXIP is an important biomarker for the detection of ovarian cancer. It is well known that ovarian cancer is the most lethal malignant tumor in gynecology. The diagnosis usually occurs in the late stage, with a 5-years survival rate of less than 30% [23]. The incidence of ovarian cancer is increasing year by year, and a large amount of data verify that there is a clear relationship between the prognosis of ovarian cancer and early detection. Early diagnosis of ovarian cancer is the primary measure that needs to be taken to reduce the mortality rate; therefore, in the field of gynecologic oncology, the screening of ovarian cancer is of great interest. In this study, we postulate that HBXIP may be used as a tumor marker, combined with clinical symptoms and signs, to detect high-risk groups and to improve the early clinical diagnosis of ovarian cancer, which may be have distinct value.

## Conclusion

To summarize, our study provides evidence that high HBXIP expression is correlated with the development of ovarian cancer. This is the first study evaluating the relationship between HBXIP expression and clinicopathological features of ovarian cancer patients, and it is of great significance to screening and targeted therapy of ovarian cancer.

## Additional file

Additional file 1: The actual survival months of each group. (DOCX 76 kb)

## Abbreviations

HBXIP: Mammalian hepatitis B X-interacting protein
IF: Immunofluorescence
IHC: Immunohistochemical
OS: Overall survival
