# Supplementary material for: HBXIP overexpression is correlated with the clinical features and survival outcome of ovarian cancer
Source: J Ovarian Res. 2017 Apr 7;10:26. doi: 10.1186/s13048-017-0322-7 (PMC5384129; doi:10.1186/s13048-017-0322-7)
Supplement: Additional file 1: — The actual survival months of each group. (DOCX 76 kb) [file 13048_2017_322_MOESM1_ESM.docx]

| **Additional file**  **Table S：The actual survival months of each group** | | | |
| --- | --- | --- | --- |
| **HBXIP (Low)** | **OS (months)** | **HBXIP (High)** | **OS (months)** |
| 1 | 15 | 1 | 3 |
| 2 | 29 | 2 | 6 |
| 3 | 36 | 3 | 22 |
| 4 | 39 | 4 | 22 |
| 5 | 40 | 5 | 23 |
| 6 | 44 | 6 | 25 |
| 7 | 45 | 7 | 25 |
| 8 | 45 | 8 | 27 |
| 9 | 48 | 9 | 27 |
| 10 | 48 | 10 | 27 |
| 11 | 49 | 11 | 30 |
| 12 | 53 | 12 | 32 |
| 13 | 54 | 13 | 32 |
| 14 | 55 | 14 | 33 |
| 15 | 62 | 15 | 34 |
| 16 | 62 | 16 | 34 |
| 17 | 64 | 17 | 34 |
| 18 | 65 | 18 | 35 |
| 19 | 65 | 19 | 35 |
| 20 | 66 | 20 | 35 |
| 21 | 67 | 21 | 37 |
| 22 | 68 | 22 | 38 |
| 23 | 68 | 23 | 38 |
| 24 | 72 | 24 | 41 |
| 25 | 72 | 25 | 41 |
| 26 | 74 | 26 | 41 |
| 27 | 74 | 27 | 44 |
| 28 | 79 | 28 | 45 |
| 29 | 82 | 29 | 45 |
| 30 | 82 | 30 | 46 |
| 31 | 83 | 31 | 46 |
| 32 | 87 | 32 | 48 |
| 33 | 87 | 33 | 49 |
| 34 | 91 | 34 | 49 |
| 35 | 93 | 35 | 50 |
| 36 | 95 | 36 | 50 |
| 37 | 101 | 37 | 53 |
| 38 | 102 | 38 | 53 |
| 39 | 103 | 39 | 53 |
| 40 | 104 | 40 | 53 |
| 41 | 107 | 41 | 54 |
| 42 | 107 | 42 | 54 |
| 43 | 111 | 43 | 56 |
| 44 | 113 | 44 | 56 |
| 45 | 116 | 45 | 57 |
| 46 | 119 | 46 | 58 |
| 47 | 120 | 47 | 59 |
| 48 | 120 | 48 | 59 |
|  |  | 49 | 62 |
|  |  | 50 | 62 |
|  |  | 51 | 65 |
|  |  | 52 | 65 |
|  |  | 53 | 69 |
|  |  | 54 | 69 |
|  |  | 55 | 69 |
|  |  | 56 | 72 |
|  |  | 57 | 72 |
|  |  | 58 | 73 |
|  |  | 59 | 74 |
|  |  | 60 | 75 |
|  |  | 61 | 78 |
|  |  | 62 | 83 |
|  |  | 63 | 92 |
|  |  | 64 | 94 |
|  |  | 65 | 94 |
|  |  | 66 | 95 |
|  |  | 67 | 98 |
|  |  | 68 | 99 |
|  |  | 69 | 100 |
|  |  | 70 | 104 |
|  |  | 71 | 115 |
|  |  | 72 | 118 |
